# Treatment of metastatic lesions localized in the acetabulum

**DOI:** 10.1186/s13018-016-0384-z

**Published:** 2016-04-28

**Authors:** Grzegorz Guzik

**Affiliations:** Department of Orthopedic Oncology, Podkarpacie Oncology Centre, Specialist Hospital in Brzozów, Bielawskiego 18, 36-200 Brzozów, Polska

**Keywords:** Bone metastases, Bone cement augmentation, Pelvic surgeries, Acetabuloplasty, Modular endoprosthesis

## Abstract

**Background:**

Metastatic lesions localized in the periacetabular area cause troublesome pain and reduced mobility of the patients. Radiotherapy effectively decreases pain, yet it does not restore the ability to load the joint. Surgical treatment involving resection of metastatic lesions and joint reconstruction using bone grafts is burdened with a high rate of complications. Modular tumor prostheses are being increasingly used. In some cases, it is possible to strengthen the acetabular roof with bone cement using vertebroplasty kits. The aim of the study was to demonstrate various methods of treatment of metastatic lesions localized in the periacetabular area together with the analysis of their results and effectiveness.

**Methods:**

Between 2010 and 2015, 27 patients with cancer metastases to the acetabulum were treated at our department. Qualification for surgical treatment was multifaceted with numerous aspects being considered. They included patients’ general condition, type of neoplasm, clinical stage, and prognosis. CT and MRI scans of the pelvis were performed in each case. Before the surgery and 3 months following the surgery, visual analogue scale (VAS) pain intensity, Karnofsky functional status, and motor ability according to the Harris scale were evaluated. Bone cement (PMMA)-augmentation was performed in 21 patients, of whom nine had cement injected precutaneously and 12 at proximal femur resection alloplasty. Hemipelvectomy Type II combined with implantation of LUMiC resection prosthesis of the acetabulum were performed in six cases.

**Results:**

The quality of life improved in all the patients. After percutaneous cement injection, the mean pain intensity VAS score was 2.7, and the mean Karnofsky functional status score was 71.8. The mean postoperative Harris hip score (HHS) was 94 points. The patients who had undergone resection alloplasty on the proximal femur combined with periacetabular cement injection were walking using one crutch. In this group of patients, the mean postoperative pain intensity, functional status, and gait efficiency scores were 4.5, 65.7, and 82 points, respectively. The mean pain intensity VAS score in patients who had LUMiC prostheses implanted was 3.4. Their mean functional status score was 65 and the gait efficiency score 71 points. All the patients were able to walk on crutches.

**Conclusions:**

Strengthening of the acetabular roof with bone cement in a specific group of patients is an adequate method of treatment which decreases pain and allows for loading the affected limb while walking. Internal hemipelvectomy combined with LUMiC prosthesis implantation makes it possible for the patients to walk using crutches and significantly reduces pain.

## Background

Bone metastatic lesions concern between 30 and 70 % of patients. The sites most commonly affected are the spine, the pelvic bones, and the proximal part of the femur and the humerus. Lytic and mixed metastases may cause tormenting pain, reduced mobility, and pathological fractures. Massive metastases to the pelvis, especially to the periacetabular area, are still a difficult treatment problem. They inhibit patients’ walking independently, thus forcing necessity to use crutches or a walking frame. Joint motion is usually significantly limited and related to pain. These infirmities are accompanied by imminent muscle atrophy. Bed-ridden patients are more likely to develop thromboembolism and infectious complications. The patients need constant care of the family or healthcare practitioners and require analgesic treatment [1–3].

Until recently, the treatment of metastatic lesions localized in the pelvis was limited to radiotherapy which effectively decreased pain but did not restore the ability to load the affected joint [2].

Treatment planning requires an in-depth analysis of patient’s general condition, stage of cancer, and prognosis. Qualification must be multifaceted and multidisciplinary. It is necessary to perform CT and MRI so as to carefully examine the extent of the lesions, infiltration of muscles, vessels, and nerves, and to plan bone reconstruction. The use of massive bone grafts to reconstruct losses in the acetabular area is controversial, because of high risk of infections and bone healing problems. Specially designed modular prostheses, the acetabular part of which is seated in the iliac ala or even in the lumbar vertebra, are being increasingly used. In the case of minor lytic lesions when the cortical bone is undamaged, it is a good solution to perform PMMA-augmentation with the use of vertebroplasty kits. Bone cement increases the resistance of the acetabulum and allows full loading of the affected limb. In some cases, it is possible to seat the acetabular part of the hip prosthesis without the need for complicated reconstructions [4–10].

### Aim of the study

The aim of this study was to demonstrate various methods of treatment of tumor-induced losses localized in the periacetabular area together with the analysis of their results and effectiveness.

## Methods

Between 2010 and 2015, 27 patients with cancer metastases to the acetabulum were treated at the orthopedic oncology department in Brzozów. The following aspects were analyzed in the qualification for the treatment: patient’s general condition, type of cancer, clinical stage of cancer, and prognosis. Each time, CT and MRI were carried out prior to the surgery. What were analyzed were the extension of bone losses, the cortical bone condition, and the possibility of hip replacement. Before the surgery and 3 months following the surgery, visual analogue scale (VAS) pain intensity, Karnofsky functional status, and motor ability according to the Harris scale were evaluated. The analysis covered the course of the surgery and the perioperative period and complications and their causes. Follow-up radiographs were performed on postoperative day 1 and then on a 6-week basis. Careful attention was given to whether there were symptoms of local recurrence of the neoplastic disease, new foci of the disease, damage to implants, or their loosening.

**Fig. 1:**
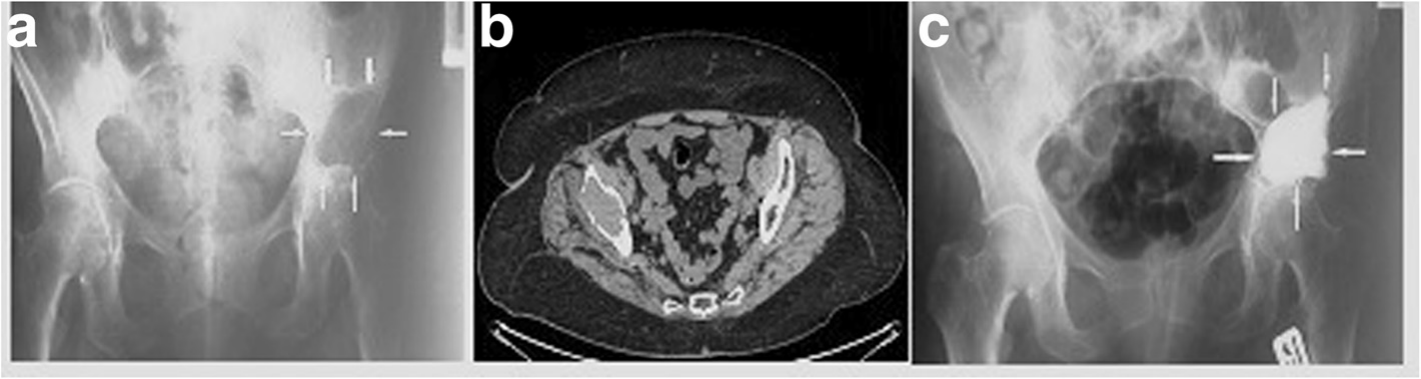
Anteroposterior radiogram (**a**) and transverse CT scan (**b**) in a kidney cancer patient with lytic bone loss in the right periacetabular area. Follow-up radiogram after bone cement (PMMA) augmentation (**c**)

**Fig. 2:**
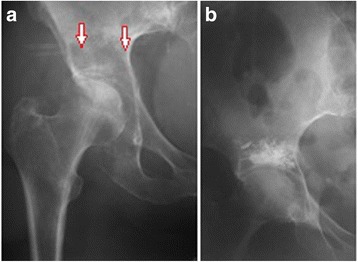
Anteroposterior radiogram (**a**) with lytic bone loss in the right periacetabular area and radiogram after bone cement (PMMA) augmentation (**b**)

**Fig. 3:**
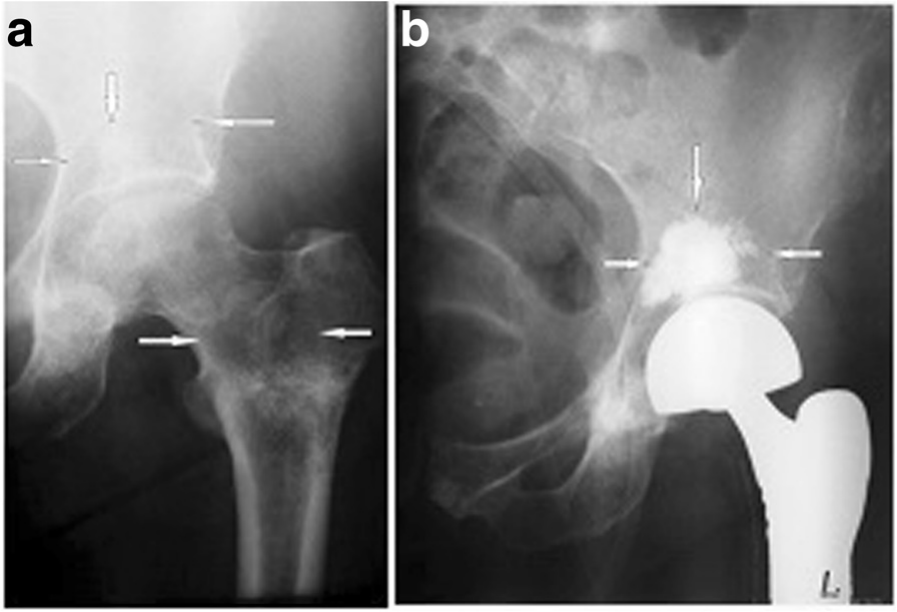
Anteroposterior radiogram of the pelvis in a patient with breast cancer metastasis to the proximal femur and the acetabulum (**a**). Postoperative radiogram (**b**). Visible resection prosthesis of the hip joint and methacrylate (PMMA) that was used for reconstruction of bone loss in the acetabulum

**Fig. 4:**
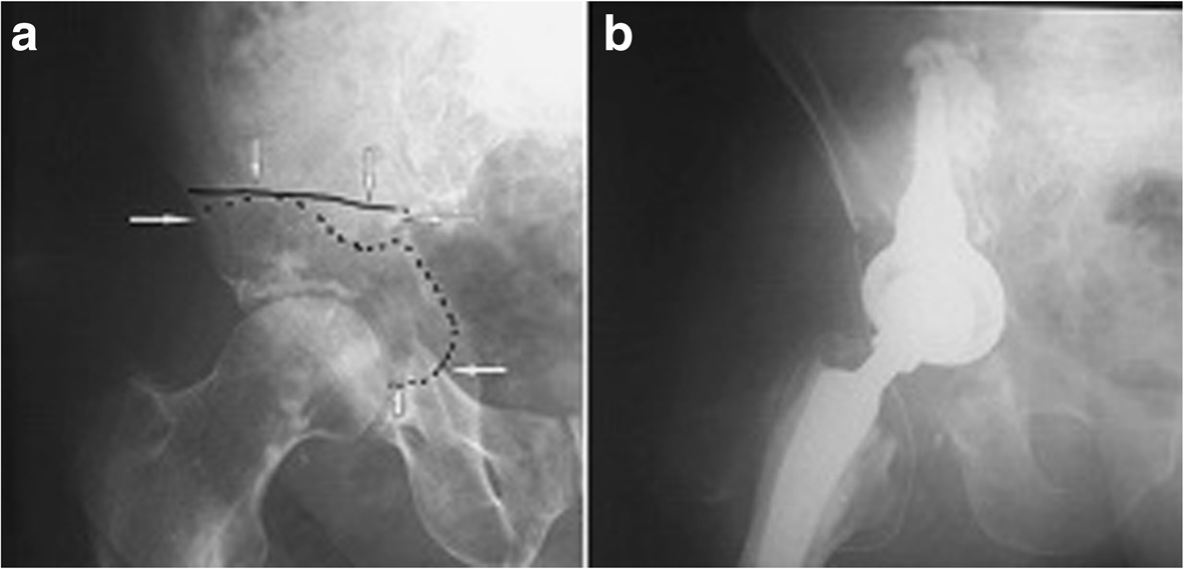
Preoperative (**a**) and postoperative radiogram (**b**) in a patient after resection of a metastatic tumor in the pelvis. Internal hemipelvectomy type II was performed (the size of metastasis and the extension of the resection of the hip bone was marked with a highlighter); a LUMiC prosthesis was implanted with the use of cement. A standard stem of hip prosthesis was implanted after resection of a metastasis localized in the neck of the femur

**Fig. 5:**
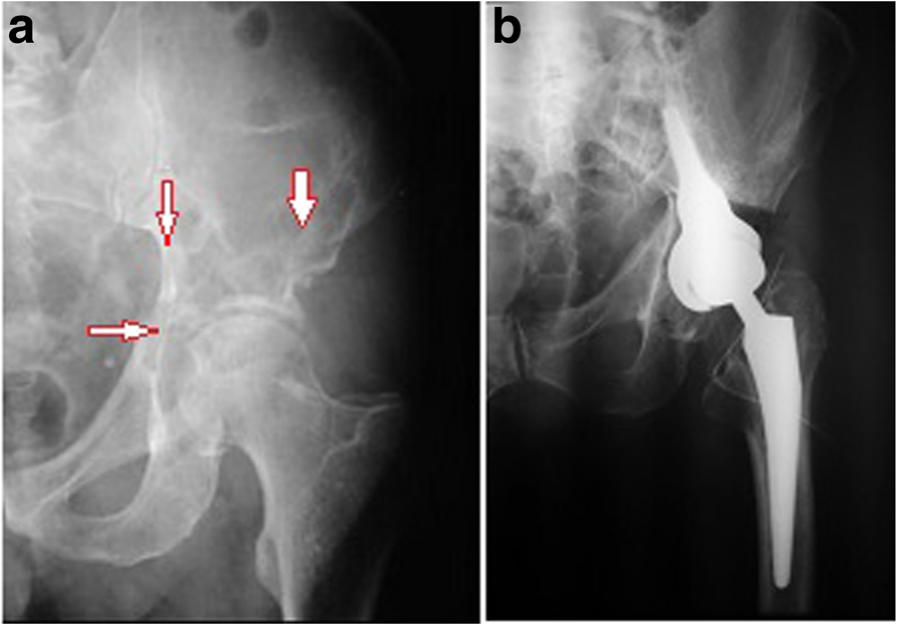
Preoperative radiogram of a metastatic tumor in the pelvis (**a**) and after LUMiC endoprosthesis implantation (**b**)

Bone cement (PMMA) augmentation was performed in 21 patients, of whom nine had cement injected precutaneously (Figs. 1 and 2), while 12 patients required proximal femur resection alloplasty, in which case the cement was injected using an open surgery method (Fig. 3). Each time, a full volume of 10 ml of bone cement was given in a single injection to prevent leakage. The cement was infused within approximately 5 min using titration method, several times changing the position of a needle in the hip bone. Metastatic tumor was resected in six patients (hemipelvectomy type II), and a LUMiC resection prosthesis of the acetabulum was implanted (Figs. 4 and 5). Two prostheses were implanted without cement, while four prostheses were implanted using vacuum-mixed bone cement injected with a positive pressure.

Three weeks after the surgery, all the patients were subjected to a single radiation dose of 8 Gy. The observation period lasted between 4 and 49 months (mean 16 months).

The majority of the treated patients (18) were women. There were nine men. The mean age was 63 for women (range 42–81) and 68 for men (range 54–82).

In our study, the most common cause of the destruction of the acetabulum was a metastasis from breast cancer (11 patients), followed by multiple myeloma (seven patients), kidney cancer (six patients), and lung cancer (three patients). The mean time interval between the diagnosis of cancer and the occurrence of a metastasis was 22 months.

The research has been performed in accordance with the declaration of Helsinki. As this retrospective analysis consists of anonymised clinical routine data, the Research Ethics Committee (Okręgowa Izba Lekarska in Crakov, ul Krupnicza, 11a 31-123) deems the application for and issue of an Ethics approval not necessary. All the patients gave a written consent to the use of data for research.

## Results

The patients who had undergone resection alloplasty on the proximal femur combined with acetabular cementation were walking with one crutch. The pain they experienced did not increase when loading the operated limb. In this group of patients, the mean preoperative and postoperative VAS pain intensity scores were 8.2 and 4.5, respectively. The mean Karnofsky functional status score was 47.2 before the surgery and 65.7 after the operation. The postoperative gait efficiency score (HHS) was 82 points.

In the group of patients with LUMiC prostheses, the mean preoperative VAS pain intensity score was 8.1 and the mean preoperative Karnofsky functional status score was 40.2. The following mean scores were recorded after the surgery: VAS 3.4, Karnofsky 65, HHS 71 (Table 1).

**Table 1:** The table presents the mean pain intensity, functional status, and gait efficiency scores in pre- and postoperative periods

Type of surgery	VAS	VAS	Karnofsky	Karnofsky	HHS
	Before the surgery	After the surgery	Before the surgery	After the surgery	After the surgery
PMMA	6.9	2.7	52.5	71.8	94
PMMA + PFE	8.2	4.5	47.2	65.7	82
LUMiC	8.1	3.4	40.2	65	71

Abbreviations: *PMMA* bone cement, *PFE* proximal femur endoprosthesis

All the surgically treated patients reported improvement in the quality of life after the operation. Three patients were walking on one crutch, two of them were using two crutches, and one patient was walking unaided.

A decrease in the strength of muscles in the operated limb was noted after implantation of both LUMiC and proximal femur prostheses. Trendelenburg’s sign was positive, which was indicative of gluteal muscle dysfunction. Eight patients were able to manage the stairs alternating feet, while ten patients were reverting to each step. Until the present day, pathological fractures or local recurrences in the proximity of the operated joint have not been detected. Persisting serous discharge from a surgical wound was noted in one patient after LUMiC prosthesis implantation. Within 3 weeks, the wound healed after antibiotic treatment and there was no need for surgical management. No thromboembolic complications, damage to implants, or dislocation of prostheses were observed. Limb length discrepancy ranging from 1 to 4 cm (mean 2 cm) was not particularly troublesome. It was compensated using insoles for shoes.

Results of the treatment of bone losses in the pelvis with bone cement (PMMA) using vertebroplasty kits are very encouraging. No inflammatory reactions in soft tissues after precutaneous injection of cement were noted. Follow-up radiographic results using this method were found satisfactory. No significant degrees of leakage of bone cement into the muscles or the hip joint were observed. Further radiograms did not reveal osteolysis in the area of bone cement, bone cement dislocation, or loosening within the hip bone. No fractures within the strengthened acetabulum were noted in any of the patients. The quality of life improved in all the patients as a result of reduced pain or its complete regression. The mean VAS pain intensity score in patients undergoing isolated acetabular cementation was 6.9 before the surgery and 2.7 after the operation. The mean Karnofsky functional status score was 52.5 before the surgery and 71.8 after the procedure. The mean postoperative Harris hip score (HHS) was 94 points. All the patients were walking unaided; loading the operated limb did not cause pain.

## Discussion

The treatment for metastatic lesions localized in the periacetabular area depends on the patient’s general condition, prognosis, and the extension and localization of the lesion [11, 12].

Non-surgical treatment is employed in patients in a poor general state of health who are not considered for surgery and in patients with a tumor localized in zones I and III according to the Enneking and Dunham classification. Radiotherapy is the most frequent method of treatment that reduces pain and rate of local recurrence. Radiotherapy is also used as adjuvant therapy after surgery. Embolization, which reduces tumor size and vascularization and causes tumor calcification, is another increasingly used method. Its analgesic effect lasts for half a year. Preoperative embolization reduces intraoperative bleeding, allows for a precise tumor dissection, and shortens the time of surgery. Preoperative embolization is particularly indicated for patients with hypervascular tumors (renal and thyroid metastases).

In the treatment of prostate and breast cancers and myeloma, multiplex bisphosphonates are used. They are effective in reducing pain and the number of subsequent fractures.

Other less common treatment methods include electrochemotherapy, radiofrequency ablation, and thermotherapy [13].

Surgical treatment most often is required in patients with tumor localized in zone II according to the Enneking and Dunham classification. Wide resection is used in patients with an extensive bone loss and in solitary bone metastasis, especially from breast, thyroid, and kidney cancers. Intralesional resection is performed in patients with diffuse pelvic involvement when wide resection is not possible. Ruggieri et al. have not demonstrated any statistically significant differences in the survival to death and survival to local recurrence between the patients after wide resection and the patients after intralesional resection [14].

There are many ways of pelvic bone losses reconstruction which aim at achieving a good functional treatment outcome. The indications for different treatment methods depend on patient’s prognosis, tumor location and size, and experience and preferences of the surgeon.

In the case of pelvic bone losses due to cancer, the use of massive bone grafts is debatable as burdened with a high risk of infectious complications and healing disorders. The patients are forced to unload the operated limb. For that reason, modular or custom-made prostheses are being increasingly used.

Stem acetabular prostheses are particularly often used in patients with a good prognosis and solitary bone metastases. Patients in IV class according to the Harrington classification are candidates for this procedure. It is a relatively simple surgical technique, but instability or dislocation of a prosthesis may occur [11, 12, 15, 16].

In the Harrington I class, total hip replacement with or without acetabular support rings has been described. Very extensive bone loss requires custom-made endoprosthetic replacement.

The Harrington technique of pelvic bone loss reconstruction requires a wide surgical approach and is associated with the risk of complications; however, it guarantees a good functional outcome. It is particularly often applied in patients in Harrington III class [11, 12].

The injection of cement under pressure using vertebroplasty kits is to a certain extent a novelty. This method was applied and described by Georgy in 2009. He operated on 12 patients with lytic metastases to the hip bone and assessed the gait efficiency as well as the VAS pain intensity. The results were good. The mean preoperative VAS score was 8.6, while the mean postoperative VAS score was 3.8 [16]. Good treatment results were also accomplished by Hierholzer et al. and Yong-il Kim et al. [17, 18]. Maccauro et al. performed acetabuloplasty in 25 patients, five of whom underwent bilateral surgery. He did not observe any serious complications; symptoms of vein thrombosis occurred in two patients. Complete pain regression was recorded in the majority of patients [19].

Weil et al. succeeded in achieving positive results as regards pain which was reduced or erased in 83 % of cases in the group of 18 patients who had undergone reconstructive operation with the use of cement. The patients were able to walk unaided without taking analgesics [10].

The LUMiC prosthesis was designed to treat bone damage that occurs during the course of the neoplastic disease in the periacetabular area. The classical method involves prosthetic implantation with no cement used. The specially shaped stem has the purpose of minimizing the rotating motion of the prosthesis in relation to bones. The surface of the prosthesis allows for growing of bones into it and secondary stabilization. It is also agreed to implant the acetabulum using bone cement, which ensures the primary stability of the prosthesis and facilitates immediate limb loading [5, 20, 21].

As Capanna et al. see it, prosthetic implantation after resection of cancer metastases to bones can be performed with the use of cement which enables immediate and full limb loading. The additional merit of this method is that it reduces the risk of implant loosening after radiotherapy [22, 23].

Similarly, Hoffmann et al., Grosheger et al., and Gebert et al. studies have demonstrated that the results of the treatment of bone metastases using cement-retained prostheses are good. The risk of their loosening in consequence of radiotherapy is low, and the incidence of infectious complications is similar when cement is not used. The patients can start walking, fully loading the operated limb immediately after the surgery. In the case of cement-free prostheses, full limb loading is recommended only after 6–12 weeks following the surgery [24–26].

Some researches have shown a low rate of infectious complications (5.4–7.9 %) and aseptic loosening (5.4–8,7 %) among patients who had cement-retained prostheses implanted [23].

Saddle prostheses have failed to meet the hopes they evoked. They loosened quickly or were primarily unstable. The incidence of dislocation was high; they also caused troublesome pain. Hip joint motion and the function of the limb were limited [27]. Wedemayer and Kauther point out that functional results are unsatisfactory in patients with saddle prostheses. In 70 % of the cases, trouble walking even on both crutches was reported [28].

Wang et al. succeeded in achieving satisfactory functional results evaluated by MSTS in the group of 50 patients who had tumor prostheses implanted after resection of pelvic tumors. Despite that the center of joint rotation was moved superiorly and medially, the function of gluteal muscles was proper enough to allow good performance in walking. In 80 % of the cases, no symptoms of loosening were detected 3 years after the surgery [8].

In our study group, six patients with LUMiC prostheses reported significant pain relief and their functional status improved. A large decrease in the strength of gluteal muscles was not perceived as particularly troublesome by the patients. No infectious complications, loosening and dislocation of implants, or local recurrences of the neoplastic disease were noted.

The patients who had undergone cement augmentation appreciated a significant improvement in the quality of life. The pain significantly decreased, and the functional status improved. All the patients were able to walk unaided, fully loading the operated limb.

Patients qualified for cement augmentation of the periacetabular area should be those with without very extensive bone losses and undamaged cortical layers which allows injecting cement under positive pressure. A correct titration cementing technique reducing the risk of uncontrollable leakage is of importance [29].

## Conclusions

1. Augmentation of tumor-induced losses in the periacetabular area is an adequate method of treatment which decreases pain and allows for loading the affected limb while walking.
2. A condition necessary for qualification for the bone cement augmentation is undamaged cortical bone layer which enables injecting cement under positive pressure and prevents its leakage.
3. The LUMiC prosthesis is a good solution for the patients who require resection of the periacetabular pelvic bone. The patients maintain walking ability with the use of crutches and they experience significant pain relief.

## Abbreviations

PMMA: polymethyl methacrylate
PFE: proximal femur endoprosthesis
VAS: Visual Analoque Scale
